# A computational study of the inhibition mechanisms of P-glycoprotein mediated paclitaxel efflux by kinase inhibitors

**DOI:** 10.1186/s12918-017-0498-x

**Published:** 2017-11-21

**Authors:** Joe Bender, Jianwen Fang, Richard Simon

**Affiliations:** Biometric Research Program, Division of Cancer Treatment and Diagnosis, National Cancer Institute, 9609 Medical Center Dr, Rockville, MD 20850 USA

**Keywords:** Cancer drug resistance, Mass action kinetic modeling, Docking, Simulation, Molecular pump

## Abstract

**Background:**

Drug resistance mediated by P-glycoprotein (P-gp) renders many cancer therapies ineffective. One P-gp substrate is the widely used chemotherapy drug paclitaxel. Co-administration of paclitaxel and another drug that inhibits P-gp may enhance the therapeutic effectiveness of paclitaxel by preventing its efflux from tumor cells.

**Results:**

Here we present a computational approach that combines docking studies with mass action kinetic modeling to investigate how kinase inhibitors may inhibit P-gp mediated paclitaxel efflux. The results show that the inhibition can be attributed to competition between paclitaxel and a tyrosine kinase inhibitor (TKI) for the substrate binding domain (SBD) as well as competition between the kinase inhibitor and ATP for the nuclear (ATP) binding domain (NBD). The relative scales of these two competitions are TKI dependent and determined by the relative affinities of paclitaxel and TKIs to the SBD and NBD of P-gp, and their membrane partition coefficients. Additional simulations suggested that there is no single strategy to further improve the ability of TKIs to inhibit paclitaxel efflux and the most efficient way likely depends on the properties of the TKIs.

**Conclusions:**

The developed model fits existing experimental results well and thus detailed analyses of isolated parameters provide insight into the mechanisms of rather important drug efflux. It can be used to guide how to design better TKIs or develop feasible drug combination strategies for targeting P-gp induced drug resistance.

**Electronic supplementary material:**

The online version of this article (10.1186/s12918-017-0498-x) contains supplementary material, which is available to authorized users.

## Background

Resistance to chemotherapy and targeted therapies remains a major problem to overcome in current cancer research because most cancers inevitably develop resistance to the treatment. A common resistance mechanism in cancer is the efflux of therapeutic agents from the cell via molecular pumps such as P-glycoprotein (P-gp) [1]. P-gp is expressed in many cancers and its level may increase significantly after one or more rounds of chemotherapy. It belongs to a class of multidrug resistance (MDR) transporters with poly-specificity for hundreds of molecules ranging in size from 300 to 4000 Da. One P-gp substrate is the widely used chemotherapy drug paclitaxel. Co-administration of paclitaxel and another drug that inhibits P-gp may enhance the therapeutic effectiveness of paclitaxel by preventing its efflux from tumor cells.

P-gp is a trans-membrane protein with two nucleotide (ATP) binding domains (NBD) in its cytoplasmic region and a helix bundle to form a substrate binding domain (SBD) in its trans-membrane region [2]. The mechanism of action of P-gp is analogous to a molecular vacuum cleaner [1]. First, substrates partition into the membrane and enter the P-gp SBD. An ATP molecule then binds to the NBD in the cytoplasm and its subsequent hydrolysis provides energy to shift P-gp conformation to a position allowing the substrate to be excreted from the cell. ADP, the ATP hydrolysis product, is released and the P-gp conformation is reset to allow the process start again.

**Fig. 1:**
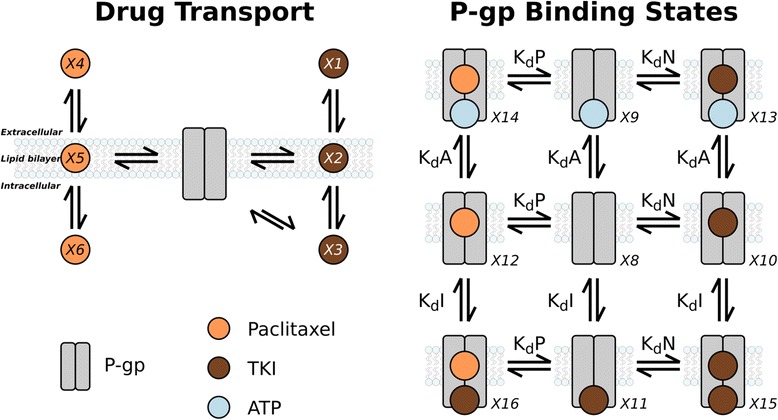
Schematic of computational kinetic model. The primary mechanism for drugs to enter the cell is via passive transport (left panel). Association of both paclitaxel and TKIs with the substrate-binding domain (SBD) occurs within the plasma membrane. Tyrosine kinase inhibitors (TKIs) interact with the nucleotide-binding domain (NBD) in the cytoplasm. Transitions between the nine distinct states of P-gp are governed by four dissociation constants (right panel): K_d_P for paclitaxel binding to the SBD, K_d_N for TKI binding to the SBD (“N” denotes nilotinib), K_d_A for the binding of ATP to the NBD, and K_d_I for TKI binding to the NBD (“I” denotes inhibitor). The values of these dissociation constants were derived from the affinities from the docking simulations and experimental data. Only the ATP-bound states lead to efflux of paclitaxel and TKIs

The NBD of P-gp shares structural similarities with NBDs in other ATP-binding proteins, including kinases; thus, many tyrosine kinase inhibitors (TKIs) are potentially capable of inhibiting P-gp. Nevertheless, these drugs are also possible substrates of the protein and therefore could be pumped out of the cell even before they reach the ATP binding domains (Fig. 1). There have been contradictory reports whether various TKIs can play roles as substrates and/or inhibitors [3]. In this study, we developed a mass action kinetic model to elucidate the details of the mechanism how co-application of TKI and paclitaxel may reduce the P-gp dependent resistance. The P-gp levels in sensitive and resistant tumor cells were estimated using parameters of well-known K562 cells, a cell line derived from an erythroleukemia, and its sub-line K562-ADR. K562 is sensitive but K562-ADR is resistant to the paclitaxel treatment (relative resistance ~ 90) [4]. This sensitivity difference is attributed to the significant difference in P-gp levels in these cell lines: while the number of P-gp molecules per cell in K562 is approximate 300, it is more than 1000-fold higher in K562-ADR [5]. We used experimental data and docking simulations to estimate the binding affinities between P-gp and three TKIs (namely first generation TKI imatinib, and second generation nilotinib and dasatinib) in both NBD and SBD in P-gp. In addition, the affinity of paclitaxel in the SBD was estimated using a docking simulation. The predicted binding affinities were then used in the mass action kinetic model of drug transport and P-gp binding. Autodock Vina, the docking program used in the present study, has demonstrated a high prediction accuracy of the binding mode prediction and a low standard error of binding affinity [6]. A number of simulations of the mass action kinetic model was performed to elucidate the details how the three TKIs affect paclitaxel efflux.

## Methods

### Docking simulations

Chemical structures of the three TKIs and paclitaxel were retrieved from PubChem as sdf files and then converted to pdb files using the openbabel software program (http://openbabel.org). The crystal structure of mouse P-gp with a cyclic peptide inhibitor (PDB ID: 3G60) was downloaded from PDB database [2]. The mouse protein structure was used because its human counterpart was not available. However, the mouse P-gp sequence is very similar to the human protein (87% identity and 93% similarity) and both share very similar substrates. The substrate in the structure was removed and the structure of remaining protein chains was minimized in Chimera using its default protein minimization setting (UCSF Chimera, production version 1.10.2). In brief, steepest descent minimization (100 steps at 0.02 Å step size) was taken to remove highly unfavorable clashes, followed by 10 steps of conjugate gradient minimization (0.02 Å step size) to further minimize the energy of the structure. The three TKIs and paclitaxel were docked into the substrate-binding pocket using AutoDock Vina (AutoDock Vina version 1.1.2. http://vina.scripps.edu). Docking systems were set up and the results were analyzed in AutoDockTools (Version 1.5.6. http://autodock.scripps.edu/resources/adt).

### Kinetic model of drug transport and P-gp binding

Simulations of the passive and P-gp-mediated transport of paclitaxel and TKIs across the plasma membrane were performed using a mass action kinetic model (detailed description can be found in the Additional file 1). The model consisted of ordinary differential equations describing the dynamics of 16 state variables. These variables included the concentrations of two drugs (paclitaxel and a TKI) in three compartments (extracellular, plasma membrane, and cytosol) as well as the concentration of cytosolic ATP and nine binding configurations adopted by P-gp (Fig. 1).

We considered only physiologically relevant concentration ranges for paclitaxel, TKIs, P-gp, and ATP. Drug concentrations typically administered to cell lines range from 10^−8^ M to 10^−4^ M. Patients treated with twice-daily dose of 400 mg nilotinib showed steady-state plasma levels between ~1.7 and ~3.6 μM, while once-daily dose of 400 mg imatinib demonstrated steady-state plasma levels of between ~2 and ~6 μM, and once-daily dose of 100 mg dasatinib was at ~4 nM and 168 nM levels (trough and peak levels, respectively) [7]. The P-gp levels in the membrane in K562 and its sub-line K562-ADR resistant to paclitaxel treatment were estimated to be 0.1 μM and 100 μM based on that the numbers of P-gp molecules in K562 and K562-ADR are approximately 300 and 300,000 [5], respectively, and the radius of these cell lines was found to be ~7 μm [8]. The thickness of cell membrane is ~8 nm. ATP concentrations in tumor cells are typically 3 mM [9].

## Results

### Estimation of TKI parameters of NBD binding

Based on data describing the TKI-mediated inhibition of calcein AM efflux in cells overexpressing P-gp [10, 11], we estimated the mass action kinetic parameters describing imatinib, nilotinib, and dasatinib affinity for the P-gp NBD (Additional file 2: Table S1). We were unable to determine from the literature whether the TKIs and calcein would compete for the substrate site; to account for this uncertainty, we performed the parameter fitting in two scenarios: one in which calcein and TKIs compete for substrate binding, and one in which calcein and TKIs are able to simultaneously occupy the P-gp substrate site. While we were unable to find evidence directly supporting either of these scenarios, some P-gp substrates have been found to noncompetitively inhibit efflux of other substrates [12, 13], suggesting that the latter scenario is feasible.

**Fig. 2:**
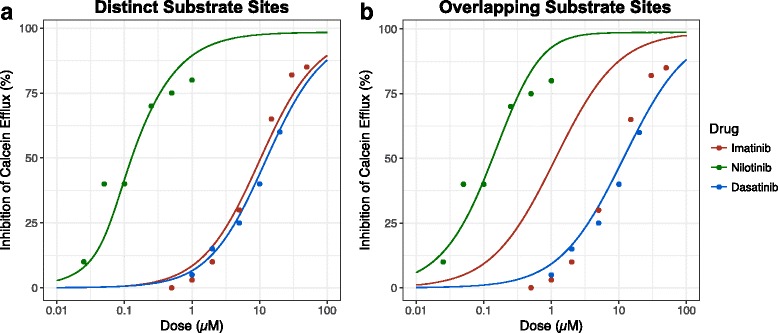
Estimation of TKI affinities to NBD. The NBD dissociation constants were estimated by calibrating the kinetic model to experimental data of TKI-mediated inhibition of calcein AM efflux in cells overexpressing P-gp. Parameter fitting was performed under two assumptions: one in which calcein and TKIs simultaneously bind to the P-gp substrate site (**a**) and one in which calcein and TKIs compete for overlapping sites (**b**)

We simulated a range of values of NBD dissociation constants for each TKI to determine the value that yielded the best fit to the experimental data (Fig. 2). The parameter fitting for each TKI dissociation constant was performed separately; since only one value was estimated at a time, we used a grid search wherein a range of potential values was tested and the one that minimized the error function was selected. The error function was the square root of the mean square error of all of the available data points. The parameter estimation conducted under the assumption that both substrates simultaneously occupy distinct sites within the P-gp substrate domain (Fig. 2a) yielded an adequate fit to the data, whereas the competitive binding assumption resulted in a poor fit for the imatinib data (Fig. 2b). The NBD dissociation constants from the noncompetitive parameter fitting were therefore used going forward: the final values for the NBD dissociation constants were estimated to be 2 nM for nilotinib, 200 nM for imatinib, and 250 nM for dasatinib.

We docked ATP into the NBD and found the predicted affinity is −7.0 kcal/mol, equivalent to a dissociation constant of 11.6 μM. Taken together, the results suggest all three drugs may act as inhibitors for the NBD with potency order nilotinib > imatinib > dasatinib, consistent with the experimental results reported by Bates et al. [3].

### Docking to substrate binding pocket

**Fig. 3:**
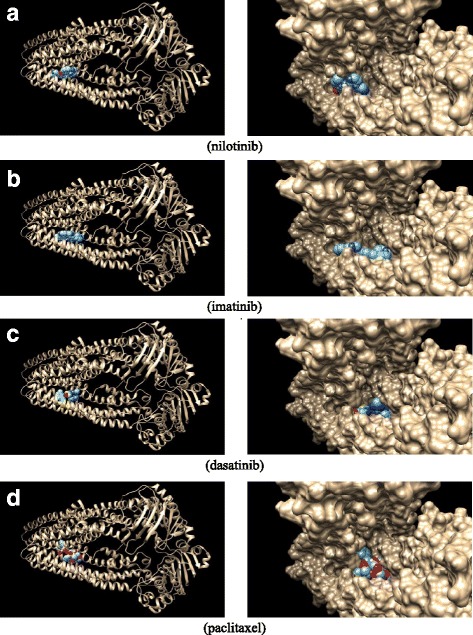
Paclitaxel and TKIs interact with the P-gp substrate-binding site. Predicted binding modes from docking studies of nilotinib (**a**), imatinib (**b**), dasatinib (**c**), and paclitaxel (**d**) in the substrate-binding pocket of P-gp, located on the left side of each panel and represented as color spheres

Docking the three TKIs into the SBD found that nilotinib was the most potent inhibitor and dasatinib was the least (Table 1). The affinity of paclitaxel to the substrate-binding site was predicted at −10.9 kcal/mol by docking, similar to nilotinib. All three TKIs and paclitaxel were predicted to bind to close locations in the pocket (Fig. 3). In addition, Fig. 3d clearly shows that the funnel-shaped substrate-binding site has sufficient space for molecules larger than paclitaxel.

**Table 1:** Predicted binding affinities for P-gp SBD based on docking studies

Drug	Predicted Binding Affinity (kcal/mol)	Partition coefficient^a^	Estimated Membrane Binding Affinity (kcal/mol)
Nilotinib	−11.1	25,704	−4.85
Imatinib	−10.3	23,988	−4.09
Dasatinib	−8.8	6606	−3.38
Paclitaxel	−10.9	3467	−5.88

^a^data were retrieved from https://www.drugbank.ca/

The binding affinities of TKIs and paclitaxel to the SBD of P-gp in the membrane were estimated from docking results and partition coefficients of TKIs and paclitaxel (Additional file 2: Figure S1). The binding affinities in the membrane are weaker than their corresponding ones in plasma because of the lipophilic environment of the membrane. Paclitaxel has relatively higher affinity in membrane than TKIs due to its relatively low partition coefficient (i.e. low lipophilic). However, the lower partition coefficient causes less accumulation of paclitaxel in the membrane than more lipophilic TKIs.

Overall, our results support the hypothesis that all three TKIs are substrates as well as inhibitors of P-gp. However, as we demonstrate in the following sections, the exact behavior of each TKI largely depends on the relative scales of the binding affinities to NBD and SBD, as well as the membrane partition coefficients. Since a TKI needs to overcome P-gp efflux before it reaches NBD, likely some of these drugs may act as inhibitors to this transporter protein only when the concentrations of these drugs are sufficiently high to pass through the membrane into the cytoplasm. These predictions are largely consistent with the experimental results reported by Bates et al. suggesting all three TKIs may overcome transporter function when their concentrations are sufficiently high [3].

### Nilotinib inhibits paclitaxel efflux more effectively than imatinib and dasatinib

**Fig. 4:**
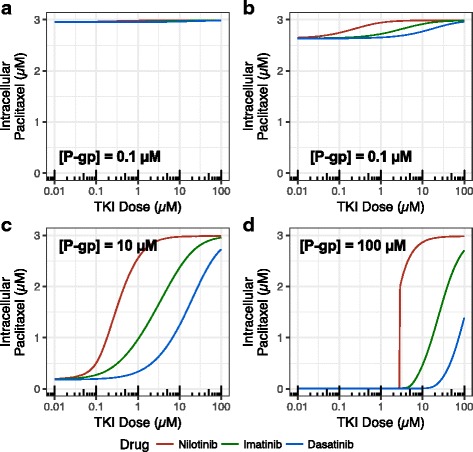
Effects of TKI concentrations on paclitaxel efflux influenced by P-gp level. Increasing doses of TKIs promote the accumulation of intracellular paclitaxel. The initial concentrations of extracellular paclitaxel and intracellular ATP were set to 3 μM and 3 mM, respectively. Higher doses of dasatinib (blue curve) were required to prevent paclitaxel efflux than imatinib and nilotinib (green and red curves, respectively). The P-gp levels were 0.1 μM (top left), 1 μM (top right), 10 μM (bottom left), and 100 μM (bottom right)

Many tumor cells are able to up-regulate P-gp expression during the development of drug resistance [14]. To analyze how the TKIs nilotinib, imatinib, and dasatinib promote the intracellular accumulation of paclitaxel, we simulated the mass action kinetics of paclitaxel and TKI transport (Fig. 4) across a range of TKI doses at four different P-gp levels: 0.1, 1, 10, 100 μM. In the models, a drug-resistant state corresponded to low intracellular concentrations of paclitaxel (i.e. P-gp is able to keep the majority of paclitaxel out of the cell). Increasing the concentration of TKI prevented P-gp-mediated resistance, leading to intracellular accumulation of paclitaxel. As expected, the P-gp level in the cell membrane played a critical role in the P-gp dependent drug resistance. At very low level of P-gp, such as 0.1 μM found in K562, almost no paclitaxel was pumped out of the cell (Fig. 4a). A level of 1 μM induced modest reductions to the paclitaxel level. At 10 μM, P-gp essentially pumped all paclitaxel back to extracellular environment. However, the efflux can be inhibited by TKIs (Fig. 4c).

The three TKIs considered in this study affected paclitaxel resistance to varying degrees. TKIs that were potent P-gp inhibitors required lower doses to achieve the same amount of paclitaxel accumulation in cytoplasm. In our simulations, nilotinib was the strongest P-gp inhibitor, while dasatinib was the weakest (Fig. 4). The high potency of nilotinib can be well explained by the fact that it has the highest affinities to the NBD and SBD and highest partition coefficient among the three TKIs. This is consistent with the experimental results of Bates et al. [3], which showed that 1 μM of nilotinib treatment was often sufficient to reverse resistance completely and low micromolar concentrations of imatinib had a partial effect while dasatinib showed only slight effects on resistance at tested concentrations [3]. At 100 μM, concentrations above clinically relevant concentration were required to increase paclitaxel in the cytoplasm (Fig. 4d).

Compared with experimental results, clearly the 10 μM is closer to the effective P-gp level than the theoretical 100 μM in K562-ADR. It can be explained by the fact that the human P-gp is less stable than the mouse protein [1]. In addition, it was found that P-gp is constitutively ubiquitinated in drug-resistant cancer cells [15]. Therefore, the effective concentration of P-gp may be lower than calculated. We used 10 μM as the effective concentration in the following simulations.

### Paclitaxel efflux at lower levels

**Fig. 5:**
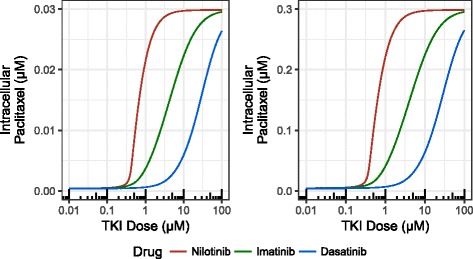
Efflux of paclitaxel at 0.03 μM (left) and 0.3 μM (right). The initial concentrations of intracellular ATP were set to 3 mM and P-gp was 10 μM

The plasma concentration of paclitaxel deceases rapidly immediately after administration but the concentration then levels off, remaining above 0.05 μM after more than 24 h [16]. To investigate how P-gp and TKI behave at lower levels of paclitaxel, we performed additional simulations at 0.03 and 0.3 μM paclitaxel (Fig. 5). Clearly, high level of P-gp (e.g. 10 μM) can easily forbid accumulation of paclitaxel in cytoplasm at these levels. As expected, potent TKI nilotinib can restore the sensitivity at sub-micromolar levels but less potent TKIs such as dasatinib may not be able to overcome the efflux at physiologically relevant concentrations for lower levels of paclitaxel.

### Mechanisms of substrate competition and ATP displacement

**Fig. 6:**
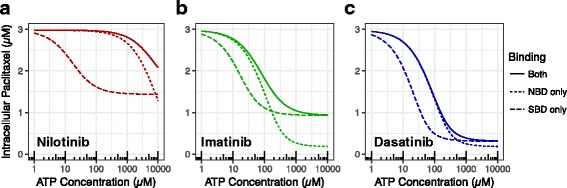
The isolated effects of the two competition mechanisms when ATP level varies. Determined by removing the binding of TKIs to the NBD and to the substrate site for nilotinib (left), imatinib (middle), and dasatinib (right). Initial concentrations of paclitaxel, P-gp, and TKI were 3 μM, 10 μM, and 1 μM, respectively. ATP concentrations were varied from 1 μM to 10 mM. The solid lines correspond to simulations where the TKI can bind to both P-gp binding sites, the dotted line corresponds to simulations where the TKI can only bind to the NBD, and the dashed line corresponds to simulations where the TKI can only bind to the substrate binding site

The kinetic scheme included two mechanisms by which a TKI could disrupt the ability of P-gp to pump paclitaxel out of the cell: direct competition with paclitaxel for the P-gp substrate-binding site within the plasma membrane, and inhibition of ATPase activity due to TKI association with the P-gp nucleotide-binding domain (NBD). To determine the relative scales of these mechanisms played in the paclitaxel efflux inhibition, we isolated the effects of each mechanism by simulating the system with the other mechanism “turned off” (equivalent to setting K_d_I or K_d_N in Fig. 1 to infinity for turning the binding of TKIs to the NBD or SBD, respectively) at a fixed TKI concentration of 1 μM. We varied the concentration of ATP to model how the energy balance of the cell affects the ATPase activity of P-gp. Figure 6 shows the intercellular levels at various ATP concentration when both mechanisms are considered (solid line), only NBD (dotted line) and only SBD (dashed line) were enabled. The relative contributions of NBD and SBD bindings were quite different among three TKIs (Fig. 6). At the normal ATP level in tumor cells (i.e. 3 mM), NBD inhibition played more important roles than SBD inhibition for nilotinib (Fig. 6a). However, a reversed order was predicted for both imatinib (Fig. 6b) and dasatinib (Fig. 6c).

In all simulations, reducing the concentration of ATP increased the intracellular level of paclitaxel (Fig. 6), consistent with the previous experimental results suggesting ATP depletion can sensitize drug-resistant cells to chemical agents [17]. In addition to the requirement of ATP as the energy source for pumping the drugs back to plasma, higher levels of ATP made it more difficult for TKI to compete against ATP for the NBD. The paclitaxel concentration when both competition mechanisms were active was higher for nilotinib than when only NBD or SBD competition was present (Fig. 6a). This indicates that nilotinib is an effective P-gp inhibitor due to its ability to bind with high affinity to P-gp at both SBD and NBD.

The difference among SBD-only curves reflects the differences between the TKIs in the two model parameters that determine the extent of SBD interactions: binding affinity for the substrate-binding site and the membrane partition coefficient. The substrate-binding site affinity and the membrane partition coefficient for dasatinib were lower than those of the other two TKIs. This makes dasatinib a less effective competitor for paclitaxel, allowing paclitaxel to be kept out of the cell by P-gp more effectively than treated with other two TKIs. The ATP level affected paclitaxel efflux in SBD-only simulations because the machinery needs ATP as energy source.

To isolate the effects of TKI association with the NBD, we simulated the model in the absence of substrate-binding site-TKI interactions (dotted lines in Fig. 6). For all the TKIs, paclitaxel concentrations were lower when the ATP concentration was higher, likely caused by the competition between TKIs and ATP for binding to the NBD. Nilotinib was the most effective TKI at displacing ATP. At low levels of ATP, the NBD binding was the dominant mechanism for paclitaxel efflux for all three TKIs. Its effect relative to SBD binding was reduced when the ATP level increased. For each of the three TKIs, there exists a point when SBD binding mechanism surpasses NBD binding one as the dominant mechanism for the paclitaxel efflux. The ATP level of the point, however, differs for each TKIs. Figure 6 clearly shows that the transition point of nilotinib (~8 mM, Fig. 6a) is higher than those of imatinib (~0.1 mM, Fig. 6b) and dasatinib (~0.4 mM, Fig. 6c).

### PGP level and paclitaxel efflux

**Fig. 7:**
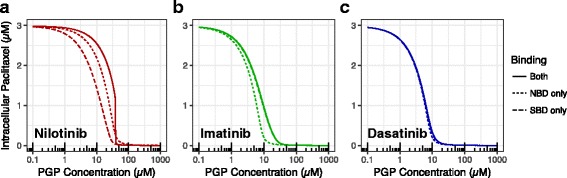
The isolated effects of the two competition mechanisms when P-gp level varies. Determined by removing the binding of TKIs to the NBD and to the substrate site for nilotinib (left), imatinib (middle), and dasatinib (right). Initial concentrations of paclitaxel, ATP, and TKI were 3 μM, 3 mM, and 1 μM, respectively. P-gp concentrations were varied from 0 to 100 μM. The solid lines correspond to simulations where the TKI can bind to both P-gp NBD and SBD, the dotted line corresponds to simulations where the TKI can only bind to the NBD, and the dashed line corresponds to simulations where the TKI can only bind to the SBD

To illustrate how the P-gp level may affect the NBD and SBD binding mechanisms for the drug resistance, we simulated NBD only, SBD only, and both mechanisms at effective P-gp levels varied from 0.1 μM to 100 μM when 1 μM TKI was presented in plasma and ATP concentration in cytoplasm was set to 3 mM (Fig. 7). Clearly, increasing P-gp level reduced intracellular paclitaxel concentration rapidly. When P-gp level reached certain level, 1 μM TKI was not able to inhibit P-gp and intracellular paclitaxel level was essentially reduced to 0. This level depended on the type of TKI and it was higher for potent TKIs such as nilotinib (Fig. 7a) than less potent imatinib (Fig. 7b) and dasatinib (Fig. 7c). For nilotinib, its bindings to both NBD and SBD contributed to efflux inhibition although NBD binding over-weighted SBD binding. The SBD binding of imatinib, however, was far more predominant than its NBD binding. The relative importance of NBD and SBD bindings were determined by the binding affinity to NBD, SBD and partition coefficient of TKIs and paclitaxel.

### Hypothetical improvement in NBD, SBD affinities and partition coefficient affect intracellular paclitaxel accumulation

**Fig. 8:**
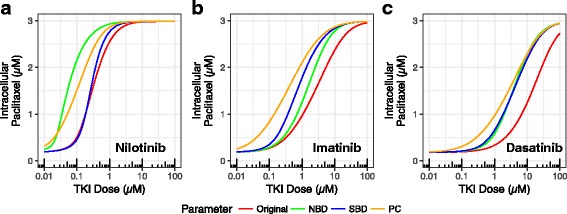
Hypothetical improvement in NBD, SBD affinities and partition coefficient (PC) affect intracellular paclitaxel accumulation. Created by hypothetically improve affinity to NBD, SBD, and partition coefficient, separately, by 10 folds for nilotinib (left), imatinib (middle), and dasatinib (right). Initial concentrations of P-gp, paclitaxel and ATP were 10 μM, 3 μM, and 3 mM, respectively

Because NBD, SBD affinities and membrane partition coefficient of a TKI may affect its ability to inhibit paclitaxel efflux, we performed simulations to predict how to further improve paclitaxel efflux inhibition. For all three TKIs used in the study, we simulated each with hypothetical 10-fold improvement in K_d_ for NBD and SBD, and partition coefficient in membrane, separately (Fig. 8). Interestingly, each TKI behaved differently in these hypothetical scenarios. For example, while nilotinib was more sensitive to NBD binding affinity improvement than SBD affinity and partition coefficient at most ATP concentrations (Fig. 8a), hypothetical higher partition coefficient would make imatinib more efficient inhibitor to paclitaxel efflux than higher NBD and SBD binding affinities (Fig. 8b). Although the partition coefficients of imatinib and nilotinib are similar, imatinib is more sensitive to the partition coefficient improvement because the mechanism of SBD binding contributed relatively more to imatinib inhibition than nilotinib (Fig. 8a, b). Changes to nilotinib SBD affinity showed little effect when the TKI level was below 200 μM to moderate improvement when its level was above 200 μM to the efflux inhibition, likely due to the its high affinity to NBD (Fig. 8a). Higher SBD affinity caused more TKI excreted back to plasma and thus reduced its intracellular level, which in turn weakened inhibition to the P-gp. For dasatinib, all hypothetical improvement resulted notable enhanced paclitaxel efflux, probably due to the relative scales of its competition against paclitaxel for the SBD and ATP Displacement should also contribute to the outcome (Fig. 8c).

Overall, the relative scales of direct competition through SBD binding and inhibition by NBD association were different for each individual TKI. It is also noteworthy that the SBD affinity is likely correlated with partition coefficient because most P-gp substrates are hydrophobic. Apparently, there is no single strategy to further improve the ability of TKIs to inhibit paclitaxel efflux and the most efficient way likely depends on the properties of the TKI.

## Discussion

Our results suggest that the two mechanisms of TKI-mediated inhibition of P-gp function occur to varying degrees, depending on the characteristics of the drugs and the concentration of P-gp and ATP in the cells. The three TKIs considered here all bind to the P-gp SBD and NBD, but nilotinib has the highest affinity in both cases. Consequently, it is the strongest inhibitor of P-gp among the three TKIs. It is noteworthy that these two mechanisms are not independent or additive to drug resistance inhibition. When a portion of TKI binds to SBD, there are two distinct effects to the levels of intracellular paclitaxel. Firstly, it competes directly with the paclitaxel efflux and therefore increases the amount of paclitaxel accumulated in cytoplasm by reducing the efflux. Secondly, the net TKI in the cytoplasm (since a portion is pumped back to plasma) will be reduced and therefore achieves less inhibition to P-gp as molecular pump, decreasing the paclitaxel accumulation in cytoplasm. TKI binding to NBD reduce paclitaxel and TKI efflux by slowing down the molecular pumper machinery. As we demonstrated in the study, paclitaxel efflux inhibition by a TKI can benefit from improving the TKI binding to either NBD or SBD (Fig. 8), although the relative efficacy may vary. Our detailed analysis can be used to guide how to design better TKIs for targeting P-gp induced drug resistance.

A clinical implication of our work relates to the dosing strategies use for chemotherapies such as paclitaxel and TKIs. While paclitaxel is generally dosed intravenously once every 3 weeks, TKIs are frequently taken orally on a daily basis. Concentrations of both are generally on the order of 100 to 1000 nM immediately after dosing [16, 18, 19]. The concentration of TKIs would be expected to remain in this range due to daily dosing, whereas the concentration of paclitaxel is maintained closer to 10 nM for an extended amount of time prior to the next dose [16]. Therefore, the concentration of TKIs in the tumor microenvironment would generally be higher than that of paclitaxel most of time. Nevertheless, it is important to maintain TKI at sufficient levels to inhibit P-gp efflux, especially when the plasma level of paclitaxel is low and it is easy for P-gp to keep paclitaxel outside of tumor cells.

An interesting implication of our simulation results is that the energy balance in a cancer cell can determine the extent of inhibition exerted by ATP-competitive TKIs: in our simulations, less potent TKIs such as dasatinib were only able to displace ATP from the NBD when ATP concentrations were relatively low. This has been observed elsewhere: the proteasome is less active at high concentrations (1 mM) than at moderate levels of ATP (50 μM to 100 μM) [20].

The ability to be a substrate of P-gp may play an important role in the overall inhibition. This is especially true for imatinib, which has higher binding affinity to SBD than NBD. Nevertheless, its ability to inhibit the NBD is reduced because the TKI is excreted out of the cell and its intracellular level is low. Thus, a better alternative might be a non-competitive TKI targeting allosteric regulation.

## Conclusions

Our approach combines multiple computational techniques: analysis of molecular-level interactions through docking simulations and analysis of protein network-level behavior through kinetic modeling. Admittedly, the model does not consider many facts that can also influence paclitaxel efflux. For example, metabolism of paclitaxel and TKIs were ignored which can be justified by time scale we have considered. We did not consider the facts that TKI may bind to kinases and other transporters may also transfer paclitaxel and TKIs. Nevertheless, our model fits existing experimental results well and thus detailed analyses of isolated parameters provide insight into the mechanisms of rather important drug efflux, which may help design better P-gp inhibitors or develop feasible drug combination strategies. Future studies could include expansion to a large set of TKIs to screen for potential interactions with P-gp.

## Additional files

Additional file 1: Kinetic model description and parameters. (PDF 131 kb)

Additional file 2: Table S1. and **Figure S1.** (PDF 373 kb)

## Abbreviations

ATP: Adenosine triphosphate
MDR: Multidrug resistance
NBD: Nuclear binding domain
PDB: Protein Data Bank
P-gp: P-Glycoprotein
SBD: Substrate binding domain
TKI: Tyrosine kinase inhibitor
