# Supplementary material for: A computational study of the inhibition mechanisms of P-glycoprotein mediated paclitaxel efflux by kinase inhibitors
Source: BMC Syst Biol. 2017 Nov 21;11:108. doi: 10.1186/s12918-017-0498-x (PMC5699195; doi:10.1186/s12918-017-0498-x)
Supplement: Supplementary file 1 — Kinetic model description and parameters. (PDF 131 kb) [file 12918_2017_498_MOESM1_ESM.pdf]

1 Model Description

We modeled the transport of TKIs and paclitaxel into and out of a cell using a system of coupled ordinary differential equations. The drugs are present in three compartments: extracellular, lipid bilayer, and cytoplasm. P-gp is a membrane-embedded transporter and therefore can access all compartments. The drugs bind the substrate pocket of P-gp within the lipid bilayer, while TKIs and ATP bind the NBD of P-gp within the cytoplasmic compartment. Bound drugs are released from the P-gp substrate pocket directly into the extracellular compartment upon ATP hydrolysis. Representing the level of each drug in each compartment, as well as ATP and all possible P-gp-containing complexes, requires 16 state variables, which are listed in Table 1.

Table 1: Variable names and initial values

Variable	Description	Initial Value
X_1	Extracellular TKI	Varies
X_2	Lipid bilayer TKI	0
X_3	Intracellular TKI	0
X_4	Extracellular Paclitaxel	Varies
X_5	Lipid bilayer Paclitaxel	0
X_6	Intracellular Paclitaxel	0
X_7	ATP	1 mM
X_8	Pgp	Varies
X_9	Pgp-ATP	0
X_{10}	Pgp-TKI	0
X_{11}	Pgp-Inhib	0
X_{12}	Pgp-Paclitaxel	0
X_{13}	Pgp-ATP-TKI	0
X_{14}	Pgp-ATP-Paclitaxel	0
X_{15}	Pgp-Inhib-TKI	0
X_{16}	Pgp-Inhib-Paclitaxel	0

1.1 Binding affinities

The aqueous phase affinities (ΔG) for drug interaction with the P-gp substrate binding site were predicted from docking studies and converted to dissociation constants (K_d^ex) using the formula $K_\mathrm{d}^\mathrm{ex} = \exp(\Delta G/RT)$ where R is the gas constant (0.001987 kcal/mol K) and T is temperature (310 K). The aqueous phase dissociation constant K_d^ex was adjusted using the drug partition coefficient K_p to derive the lipid bilayer phase dissociation constant $K_\mathrm{d}^\mathrm{mem}$ as described in Figure S1 of additional file 1. Values of each predicted affinity are given in Table S2.

To derive rate constants k_on and k_off from the $K_\mathrm{d}^\mathrm{mem}$ values, we assumed that k_on values were controlled by diffusion, thus we estimated them using the Debye-Smoluchowski equation, $k_\mathrm{on} = 4\pi a(D_1 + D_2)N_\mathrm{A}$, where a is the radius of the area of interaction between two molecules, D_1 and D_2 are the diffusion coefficients of the two molecules, and N_A is Avogadro's number. Typically the

diffusion coefficient of the smaller molecules (drugs and ATP) are much higher than those of larger proteins, therefore we only need to consider the diffusion coefficients of the smaller molecules. The diffusion of ATP in the cytoplasm, 10^{-6} cm^2/sec, was used for the three TKIs as well based on similar molecular weights. Diffusion of drugs within the lipid bilayer is expected to be slower due to the higher viscosity of the hydrophobic phase (Stokes-Einstein equation). Assuming that the ratio of lipid bilayer viscosity to cytoplasm viscosity is the same as that of octanol to water gives a diffusion coefficient for drugs in the bilayer of 10^{-7} cm^2/sec, one order of magnitude lower than that of the cytoplasm. Assuming the radius of ATP and drugs is approximately 1 nm gives k_{on} values of approximately 1/nM/sec in the cytoplasm and 0.1/nM/sec in the lipid bilayer. The k_{off} values are obtained by multiplying $k_{\mathrm{on}} * K_{\mathrm{d}}$.

1.2 Transport across lipid bilayer

The rate of transport of drugs from the extracellular space into the lipid bilayer is driven by the concentration gradient according to the following equation: $J = k_L A(X_2 - K_{\mathrm{p}} X_1)$, where k_L is the mass transfer coefficient, A is the total area of the cell membrane, K_{p} is the octanol-water partition coefficient of the particular drug, and X_2 and X_1 are the concentrations of the drug in the lipid bilayer and the extracellular space, respectively. The flux J is defined here such that a negative value indicates net flow of molecules from the extracellular space into the lipid bilayer. We used partition coefficients for each drug from DrugBank (www.drugbank.ca). Assuming a spherical cell with radius 7 μm resulted in an area A of $6 \cdot 10^{-6}$ cm^2 [1]. We found k_L using the equation $\mathrm{Sh} = k_L L/D$, where Sh is the Sherwood number, which relates mass transfer rate to diffusion rate, L is the thickness of the lipid bilayer (8 nm), and D is the drug diffusion coefficient in the lipid bilayer. In the absence of convective flows, the Sherwood number is assumed to be 2, therefore we estimated the value of k_L to be 0.2 cm/sec for drugs in the bilayer.

1.3 Efflux/ATP hydrolysis rate constant

Maximum efflux rate has been estimated by [2] to be 3 pmol/sec/(10^6 cells). This is dependent on the number of P-gp molecules present in the system in which the measurement was taken (human epidermoid carcinoma KB8-5). This measurement was not available in this cell line, but using an approximation of 100 P-gp molecules per cell, we derive $\mathrm{k_{cat}}$ as follows:

$$\begin{aligned} \mathrm{k_{cat}} &= \frac{3\ \mathrm{pmol}}{\mathrm{sec} * 10^6\ \mathrm{cells}} * \frac{10^{-12}\ \mathrm{mol}}{\mathrm{pmol}} * \frac{6.02 * 10^{23}\ \mathrm{molecules}}{\mathrm{mol}} \Big/ \frac{100\ \mathrm{molecules}}{\mathrm{cell}} \\ &\sim 10^4/\mathrm{sec} \end{aligned}$$

Table 2: Binding affinities from docking studies. The ATP-NBD interaction takes place in the cytoplasm and therefore no correction was applied to determine the lipid bilayer phase affinity.

Drug-Site	ΔG (kcal/mol)	K_d^ex (nM)	K_p	$K_\mathrm{d}^\mathrm{mem}$ (μM)
Nilotinib-Substrate	-11.1	15	$10^{4.41}$	384
Imatinib-Substrate	-10.3	55	$10^{4.38}$	131
Dasatinib-Substrate	-8.8	624	$10^{3.82}$	413
Paclitaxel-Substrate	-10.9	21	$10^{3.54}$	72
ATP-NBD	-7.0	11604	–	–

Table 3: Parameter values. Superscripts on K_{p}, k_{on}, k_{off}, and k_{cat} indicate the species, with N indicating a TKI bound to the P-gp substrate site, P indicating paclitaxel, I indicating a TKI bound to the P-gp NBD, and A indicating ATP. Section numbers refer to the preceding sections where further discussion and citations for the parameter values can be found. The $k^{\mathrm{I}}_{\mathrm{off}}$ values are derived from calibration of the model against experimental data as described in the main text (Figure 2).

Parameter	Description	Value	Section
V_{E}	Volume of extracellular medium	$2 \cdot 10^{-6}$ cm^3	–
V_{B}	Volume of lipid bilayer	$2.5 \cdot 10^{-12}$ cm^3	–
V_{C}	Volume of cytoplasm	$1.5 \cdot 10^{-9}$ cm^3	–
A	Surface area of cell	$6 \cdot 10^{-6}$ cm^2	1.2
$K^{\mathrm{N}}_{\mathrm{p}}$	Partition coefficient of TKI	$10^{4.41}$ (Nilotinib)<br>$10^{4.38}$ (Imatinib)<br>$10^{3.82}$ (Dasatinib)	1.2
$K^{\mathrm{P}}_{\mathrm{p}}$	Partition coefficient of Paclitaxel	$10^{3.54}$	1.2
k_{L}	Mass transfer coefficient	0.002 cm/sec	1.2
$k^{\mathrm{N}}_{\mathrm{on}}$	Association rate constant	0.1/nM/sec	1.1
$k^{\mathrm{N}}_{\mathrm{off}}$	Dissociation rate constant	38358/sec (Nilotinib)<br>131189/sec (Imatinib)<br>412505/sec (Dasatinib)	1.1
$k^{\mathrm{P}}_{\mathrm{on}}$	Association rate constant	0.1/nM/sec	1.1
$k^{\mathrm{P}}_{\mathrm{off}}$	Dissociation rate constant	7159/sec	1.1
$k^{\mathrm{I}}_{\mathrm{on}}$	Association rate constant	1/nM/sec	1.1
$k^{\mathrm{I}}_{\mathrm{off}}$	Dissociation rate constant	2/sec (Nilotinib)<br>200/sec (Imatinib)<br>250/sec (Dasatinib)	fitted
$k^{\mathrm{A}}_{\mathrm{on}}$	Association rate constant	1/nM/sec	1.1
$k^{\mathrm{A}}_{\mathrm{off}}$	Dissociation rate constant	11604/sec	1.1
$k^{\mathrm{N}}_{\mathrm{cat}}$	Efflux/ATP hydrolysis rate constant	10^4/sec	1.3
$k^{\mathrm{P}}_{\mathrm{cat}}$	Efflux/ATP hydrolysis rate constant	10^4/sec	1.3
$k^{\mathrm{A}}_{\mathrm{cat}}$	Efflux/ATP hydrolysis rate constant	10^4/sec	1.3

References

[1] Henslee BE, Morss A, Hu X, Lafyatis GP, Lee LJ. "Electroporation dependence on cell size: optical tweezers study". *Analytical Chemistry.* 83(11): 3998-4003, 2011.

[2] Ghauharali RI, Westerhoff HV, Dekker H, Lankelma J. "Saturable P-glycoprotein kinetics assayed by fluorescence studies of drug efflux from suspended human KB8-5 cells". *Biochim Biophys Acta.* 1278(2): 213-22, 1996.

Differential equations. A constant with subscript or superscript N indicates the parameter specifies the behavior of a TKI (N for nilotinib). A subscript or superscript of P indicates paclitaxel, A indicates ATP, and I indicates the TKI binding to the NBD (I for inhibition).

$$
\begin{aligned}
\dot{X_1} &= \frac{1}{V_E}\left[k_L A(X_2 - K_{\mathrm{p}}^{\mathrm{N}} X_1) + k_{\mathrm{cat}}^{N} X_{13} V_C\right] \\
\dot{X_2} &= \frac{1}{V_B}\left[-k_L A(X_2 - K_{\mathrm{p}}^{\mathrm{N}} X_1) - k_L A(X_2 - K_{\mathrm{p}}^{\mathrm{N}} X_3)\right. \\
&\quad \left. + V_C\left(-k_{\mathrm{on}}^{N} X_2 X_8 + k_{\mathrm{off}}^{N} X_{10} - k_{\mathrm{on}}^{N} X_2 X_9 + k_{\mathrm{off}}^{N} X_{13} - k_{\mathrm{on}}^{N} X_2 X_{11} + k_{\mathrm{off}}^{N} X_{15}\right)\right] \\
\dot{X_3} &= \frac{1}{V_C}\left[k_L A(X_2 - K_{\mathrm{p}}^{\mathrm{N}} X_3) - k_{\mathrm{on}}^{I} X_3 X_8 + k_{\mathrm{off}}^{I} X_{11}\right. \\
&\quad \left. - k_{\mathrm{on}}^{I} X_3 X_{10} + k_{\mathrm{off}}^{I} X_{15} - k_{\mathrm{on}}^{I} X_3 X_{12} + k_{\mathrm{off}}^{I} X_{16}\right] \\
\dot{X_4} &= \frac{1}{V_E}\left[k_L A(X_5 - K_{\mathrm{p}}^{\mathrm{P}} X_4) + k_{\mathrm{cat}}^{P} X_{14} V_C\right] \\
\dot{X_5} &= \frac{1}{V_B}\left[-k_L A(X_5 - K_{\mathrm{p}}^{\mathrm{P}} X_4) - k_L A(X_5 - K_{\mathrm{p}}^{\mathrm{P}} X_6)\right. \\
&\quad \left. + V_C\left(-k_{\mathrm{on}}^{P} X_5 X_8 + k_{\mathrm{off}}^{P} X_{12} - k_{\mathrm{on}}^{P} X_5 X_9 + k_{\mathrm{off}}^{N} X_{14} - k_{\mathrm{on}}^{P} X_5 X_{11} + k_{\mathrm{off}}^{P} X_{16}\right)\right] \\
\dot{X_6} &= \frac{1}{V_C}\left[k_L A(X_5 - K_{\mathrm{p}}^{\mathrm{P}} X_6)\right] \\
\dot{X_7} &= -k_{\mathrm{on}}^{A} X_7 X_8 + k_{\mathrm{off}}^{A} X_9 - k_{\mathrm{on}}^{A} X_7 X_{10} + k_{\mathrm{off}}^{A} X_{13} \\
&\quad - k_{\mathrm{on}}^{A} X_7 X_{12} + k_{\mathrm{off}}^{A} X_{14} + k_{\mathrm{cat}}^{A} X_9 + k_{\mathrm{cat}}^{N} X_{13} + k_{\mathrm{cat}}^{P} X_{14} \\
\dot{X_8} &= -k_{\mathrm{on}}^{A} X_7 X_8 + k_{\mathrm{off}}^{A} X_9 - k_{\mathrm{on}}^{N} X_2 X_8 + k_{\mathrm{off}}^{N} X_{10} \\
&\quad - k_{\mathrm{on}}^{I} X_3 X_8 + k_{\mathrm{off}}^{I} X_{11} - k_{\mathrm{on}}^{P} X_5 X_8 + k_{\mathrm{off}}^{P} X_{12} \\
&\quad + k_{\mathrm{cat}}^{A} X_9 + k_{\mathrm{cat}}^{N} X_{13} + k_{\mathrm{cat}}^{P} X_{14} \\
\dot{X_9} &= k_{\mathrm{on}}^{A} X_7 X_8 - k_{\mathrm{off}}^{A} X_9 - k_{\mathrm{on}}^{N} X_2 X_9 + k_{\mathrm{off}}^{N} X_{13} \\
&\quad - k_{\mathrm{on}}^{P} X_5 X_9 + k_{\mathrm{off}}^{N} X_{14} + k_{\mathrm{cat}}^{A} X_9 \\
\dot{X_{10}} &= k_{\mathrm{on}}^{N} X_2 X_8 - k_{\mathrm{off}}^{N} X_{10} - k_{\mathrm{on}}^{A} X_7 X_{10} + k_{\mathrm{off}}^{A} X_{13} \\
&\quad - k_{\mathrm{on}}^{I} X_3 X_{10} + k_{\mathrm{off}}^{I} X_{15} \\
\dot{X_{11}} &= k_{\mathrm{on}}^{I} X_3 X_8 - k_{\mathrm{off}}^{I} X_{11} - k_{\mathrm{on}}^{N} X_2 X_{11} + k_{\mathrm{off}}^{N} X_{15} \\
&\quad - k_{\mathrm{on}}^{P} X_5 X_{11} + k_{\mathrm{off}}^{P} X_{16} \\
\dot{X_{12}} &= k_{\mathrm{on}}^{P} X_5 X_8 - k_{\mathrm{off}}^{P} X_{12} - k_{\mathrm{on}}^{A} X_7 X_{12} + k_{\mathrm{off}}^{A} X_{14} \\
&\quad - k_{\mathrm{on}}^{I} X_3 X_{12} + k_{\mathrm{off}}^{I} X_{16} \\
\dot{X_{13}} &= k_{\mathrm{on}}^{A} X_7 X_{10} - k_{\mathrm{off}}^{A} X_{13} + k_{\mathrm{on}}^{N} X_2 X_9 - k_{\mathrm{off}}^{N} X_{13} - k_{\mathrm{cat}}^{N} X_{13} \\
\dot{X_{14}} &= k_{\mathrm{on}}^{A} X_7 X_{12} - k_{\mathrm{off}}^{A} X_{14} + k_{\mathrm{on}}^{P} X_5 X_9 - k_{\mathrm{off}}^{P} X_{14} - k_{\mathrm{cat}}^{P} X_{14} \\
\dot{X_{15}} &= k_{\mathrm{on}}^{I} X_3 X_{10} - k_{\mathrm{off}}^{I} X_{15} + k_{\mathrm{on}}^{N} X_2 X_{11} - k_{\mathrm{off}}^{N} X_{15} \\
\dot{X_{16}} &= k_{\mathrm{on}}^{I} X_3 X_{12} - k_{\mathrm{off}}^{I} X_{16} + k_{\mathrm{on}}^{P} X_5 X_{11} - k_{\mathrm{off}}^{P} X_{16}
\end{aligned}
$$
