# Supplementary material for: A computational study of the inhibition mechanisms of P-glycoprotein mediated paclitaxel efflux by kinase inhibitors
Source: BMC Syst Biol. 2017 Nov 21;11:108. doi: 10.1186/s12918-017-0498-x (PMC5699195; doi:10.1186/s12918-017-0498-x)
Supplement: Supplementary file 2 — and Figure S1. (PDF 373 kb) [file 12918_2017_498_MOESM2_ESM.pdf]

Table S1. Calcein AM efflux data.

Imatinib		Nilotinib		Dasatinib	
TKI Dose (nM)	Inhibition (%)	TKI Dose (nM)	Inhibition (%)	TKI Dose (nM)	Inhibition (%)
500	0	25	10	1000	5
1000	3	50	40	2000	15
2000	10	100	40	5000	25
5000	30	250	70	10000	40
15000	65	500	75	20000	60
30000	82	1000	80		
50000	85				

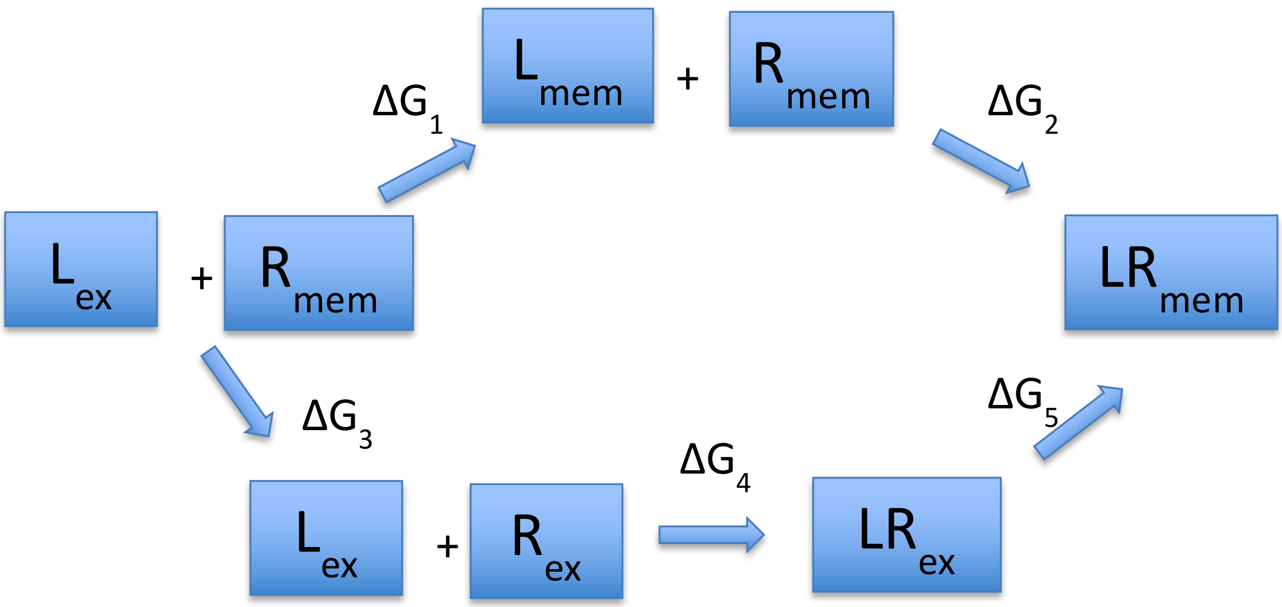

Figure S1. Theoretical basis for estimation of K_d^{mem}

$\Delta G_1 + \Delta G_2 = \Delta G_3 + \Delta G_4 + \Delta G_5$

Since: $\Delta G_3 \sim -\Delta G_5$ (ΔG_3 is the energy difference between Receptor in extracellular and membrane; instead ΔG_5 is the energy difference between ligand-receptor complex in membrane and extracellular. Since there is no interaction between ligand and the environment outside the receptor, the equation is reasonable).

Thus: $\Delta G_1 + \Delta G_2 \sim \Delta G_4$

$-RTln(K_p) + RTlnK_d^{mem} \sim RTlnK_d^{ex}$

$K_d^{mem} \sim K_d^{ex} * K_p$ also: $\Delta G_2 \sim \Delta G_4 + RTln(K_p)$

Therefore, the affinity and dissociation constant in the membrane can be estimated using docking predicted affinity and partition constant.

L: ligand; R: receptor; ex: extracellular; mem: membrane
